# Dietary Nitrogen and Its Role in the Gut Microbiome and Inflammatory Bowel Disease: A Narrative Review

**DOI:** 10.3390/nu17142373

**Published:** 2025-07-20

**Authors:** Matthew Herrera, Lauri O. Byerley

**Affiliations:** Department of Physiology, Louisiana State University Health Sciences Center, New Orleans, LA 70112, USA; mherr3@lsuhsc.edu

**Keywords:** nitrogen, inflammatory bowel disease, protein, purines

## Abstract

In recent years, gut microbiota has emerged as a critical regulator of gastrointestinal health and disease, with its role in inflammatory bowel disease (IBD)—including Crohn’s disease and ulcerative colitis—being particularly significant. Among the many factors influencing the gut microbiota, dietary components such as fibers, fats, and polyphenols have received substantial attention. However, nitrogen-containing compounds, such as amino acids, nitrates, urea, and even nucleic acids, such as purines, remain underexplored despite their integral role in shaping microbial ecology, host metabolism, and immune responses. Some of these compounds are metabolized by gut bacteria into bioactive molecules such as short-chain fatty acids, ammonia, and nitric oxide, which exert diverse effects on mucosal integrity and inflammation. IBD pathophysiology is characterized by chronic inflammation, microbial dysbiosis, and compromised epithelial barriers. Nitrogen metabolism contributes significantly to these processes by influencing microbial composition, metabolite production, and host immune pathways. The breakdown of various nitrogen-containing compounds in the body leads to the production of byproducts, such as ammonia and hydrogen sulfide, which have been implicated in mucosal damage and immune dysregulation. At the same time, nitrogen-derived molecules, such as short-chain fatty acids and nitric oxide, exhibit protective effects, underscoring the dual role of dietary nitrogen in health and disease. This narrative review highlights the complex interactions between dietary nitrogen sources, gut microbiota, and IBD pathogenesis. We summarize the mechanisms by which nitrogen compounds influence microbial dynamics, identify their contributions to inflammation and barrier dysfunction, and explore their therapeutic potential. Multidisciplinary approaches integrating clinical, metabolomic, and microbiome research are essential to unravel the full scope of nitrogen’s role in gut health and identify novel therapeutic targets.

## 1. Introduction

Inflammatory bowel disease (IBD), including Crohn’s disease (CD) and ulcerative colitis (UC), represents a set of chronic inflammatory conditions affecting the gastrointestinal tract. Globally, IBD affects approximately 0.3% to 0.5% of the population, with incidence rates increasing in newly industrialized nations [1,2]. The etiology of IBD is complex and not completely understood, but it is believed to be an interaction of genetic predisposition, environmental triggers, immune system malfunctions, and disruptions in the gut microbiome [3].

The human gut microbiome, a complex ecosystem of trillions of microorganisms, plays a critical role in maintaining intestinal health by influencing nutrient metabolism, modulating immune responses, and protecting against harmful pathogens [4]. It is also directly involved in IBD pathogenesis. Recent advancements in high-throughput sequencing technologies have provided deeper insights into the gut microbiota, revealing disruptions in the microbial composition and functions in IBD patients compared to healthy controls [5]. This dysbiosis is characterized by a reduction in beneficial bacteria, such as *Faecalibacterium prausnitzii*, and an increase in potentially harmful bacteria [6,7]. This altered microbiota can lead to a compromised intestinal barrier, allowing bacterial translocation and triggering an excessive immune response [7], characteristic of inflammatory conditions like IBD.

The gut microbiota also influences the development and function of both innate and adaptive immune cells. For instance, certain commensal bacteria promote the development of regulatory T cells, which are crucial for maintaining immune tolerance. In IBD, this balance is disrupted, leading to an overactive immune response and chronic inflammation [8,9].

Furthermore, the gut microbiota produces various metabolites that can modulate immune function. For example, short-chain fatty acids (SCFAs), produced by bacterial fermentation of dietary fiber, have been shown to have anti-inflammatory properties and support intestinal barrier function [8]. In IBD, there is often a reduction in SCFA-producing bacteria, contributing to the inflammatory state [8,10]. While overshadowed by these metabolites, dietary nitrogen compounds such as amino acids, nitrates, ammonia, and urea can also play a significant role in the pathogenesis of IBD [11], particularly due to dietary proteins and other nitrogen-containing compounds serving as substrates for gut bacteria, influencing their composition and metabolic activities [6].

Several key aspects of IBD pathology in the colon are relevant to interactions with nitrogen-containing compounds. IBD is characterized by increased intestinal permeability, allowing greater interaction between luminal contents (including nitrogen compounds) and the mucosal immune system [12]. Additionally, the protective mucus layer is often compromised in IBD, potentially increasing exposure of the epithelium to nitro-containing compounds [12]. IBD patients exhibit altered gut microbiota composition, with reduced diversity and changes in bacterial populations involved in nitrogen metabolism [13]. The inflamed colon observed in IBD patients shows increased immune cell presence, which may also interact with nitrogen compounds and their metabolites [12]. Alterations in colonic pH in IBD can affect the metabolism of nitrogen compounds by gut bacteria [14]. Finally, IBD can lead to malabsorption, potentially increasing the availability of dietary nitrogen compounds in the colon [15].

These pathological changes create an environment where nitrogen-containing compounds from the diet or produced by bacteria may have increased interactions with the colonic mucosa and immune cells, potentially influencing disease progression or symptoms in IBD. These interactions between nitrogen compounds, the gut microbiome, and the inflamed colonic environment highlight the complex pathophysiology of IBD and suggest potential avenues for further research. However, the pathways involved in nitrogen metabolism are complex and vary between individuals, and their complexities make it challenging to research nitrogen-specific metabolites like ammonia and nitric oxide [16]. Studies on the impact of nitrogen on overall gut microbiota and IBD pathogenesis vary in significance and methodology, making it challenging to summarize conclusions about the effects of dietary nitrogen on gut health, and many of the diverse sources of nitrogen in the human diet (e.g., protein-derived amino acids, nitrates in vegetables, and urea from endogenous metabolism) remained poorly characterized with respect to gut health [14].

But there is an obvious relationship between the gut microbiota, nitrogen metabolism, and pathophysiologic mechanisms associated with IBD. Nitrogen compounds, encompassing amino acids, nitrates, and urea, are essential for microbial metabolism and play a central role in gut microbiota dynamics. However, their contributions to gut health and inflammatory bowel disease (IBD) pathogenesis remain poorly understood compared to other dietary factors like fibers and fats. While the research to date has largely focused on substances like polyphenols and macronutrients like dietary fibers, there is a critical gap in our knowledge regarding the complex interactions between nitrogen metabolism, microbial ecology, and immune responses in the gut [11]. Therefore, this narrative review will instead emphasize the effects of various sources of dietary nitrogen on IBD.

**Figure 1:**
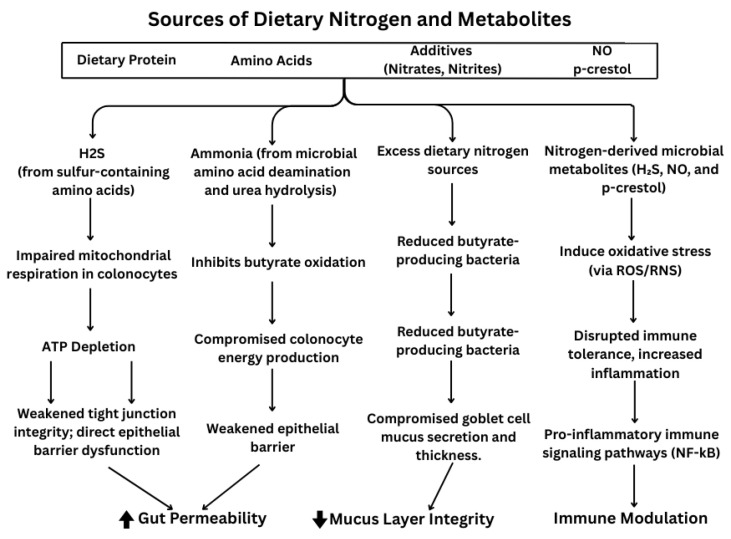
Dietary nitrogen’s effect on gut permeability, mucus layer integrity and immune modulation.

Excess nitrogen metabolism in the gut can lead to the generation of bacterial metabolites that negatively impact intestinal homeostasis. Several of these compounds, such as ammonia and *p*-cresol, for example, can interfere with colonocyte energy metabolism, primarily by impairing mitochondrial respiration and reducing ATP production, which in turn limits epithelial renewal and barrier maintenance [17]. These nitrogen-derived byproducts may also promote oxidative and nitrosative stress through the formation of reactive oxygen and nitrogen species (ROS and RNS), further contributing to epithelial damage and mucosal inflammation [18]. In particular, *p*-cresol—produced from bacterial tyrosine fermentation—has been shown to increase paracellular permeability, suppress epithelial proliferation, and induce oxidative stress in both human and animal models [19]. These combined effects—on energy metabolism, epithelial barrier integrity, and redox signaling—highlight how excessive nitrogen exposure can contribute to the inflammatory environment observed in IBD (Figure 1).

## 2. Sources of Dietary Nitrogen

### 2.1. Amino Acids

Amino acids are the main nitrogen-containing components derived from dietary proteins [20]. They are abundant in animal-based foods such as meat, fish, eggs, and dairy products, as well as plant-based sources like legumes, nuts, and grains. Amino acids are the building blocks of proteins and play a crucial role in maintaining gut mucosal integrity and supporting microbiota growth. The consumption of diverse protein sources, for example, casein (animal source) and soy (plant source), significantly impacts gut microbial metabolism, particularly amino acid degradation pathways [20]. Dietary proteins are broken down into amino acids and peptides through enzymatic digestion in the gastrointestinal tract. This process begins in the stomach with pepsin and continues in the small intestine with the action of pancreatic proteases such as trypsin and chymotrypsin. Once broken down, amino acids and peptides are absorbed in the small intestine [21]. The majority of dietary protein is absorbed in the small intestine (>90%), but the fraction that remains reaches the colon and serves as a substrate for microbial metabolism [22,23].

The diverse range of protein types in the human diet can lead to the generation of different peptides in the gastrointestinal tract, and these peptides generated from the metabolism of dietary proteins can alter the gut microbiota. The proteins from animal-based sources have been shown to have beneficial effects on gut microbiota compared to food sourced from plants, largely due to the easier ability of the human gut to digest animal proteins compared to plants [24,25]. Plant-based proteins, such as soybean and peanut, while often used in human food production, can be harder to digest due to anti-nutritional factors, such as glycinin and beta-conglycinin [26]. Plant-based foods used in vegetarian diets, while containing almost all twenty amino acids, have still been considered to be inadequate in amino acid composition due to less optimal levels of these amino acids [25]. While animal protein is easier to digest in the gut, a diverse diet including both plant- and animal-sourced nutrients has been linked to greater amounts of bacterial diversity. Prior studies have demonstrated that individuals who consumed more than 30 plant types per week, compared to those who consumed less than 10 plant types, showed increased levels of the species *Faecalibacterium prausnitzii* and genus *Oscillospira*, typically known to be short-chain fatty acid (SCFA) fermenters [27]. However, dietary research methodologies do not often account for the diversity of protein sources in diets, which could explain why there are variable outcomes associated with broad investigations of human diets. Incorporation of dietary diversity indices could provide more insight into the nuanced effects of dietary diversity on gut microbiota [27].

Amino acids like glutamine and arginine, found in foods like fish, eggs, and dairy, are particularly beneficial for gut health, aiding in immune function and maintaining epithelial barrier integrity [28]. While a large portion of dietary protein is absorbed in the small intestine, the large intestine plays an important role in absorbing amino acids derived from endogenous secretions such as mucus, shed epithelial cells, and microbial amino acids, as well as derived from dietary protein. It is estimated that about 2–7% of daily protein intake reaches the large intestine [29], and these residual amino acids and peptides that escape digestion are fermented by the gut microbiota in the colon, resulting in the production of various metabolites, such as short-chain fatty acids (SCFAs), ammonia, and branched-chain fatty acids [16,29].

Sulfur-containing amino acids, such as methionine and cysteine, have been implicated in IBD. These amino acids are primarily found in proteins such as beef, chicken, and dairy [30,31]. Proteins contain about 3 and 6% sulfur amino acids, and a small amount of sulfur comes in the form of inorganic sulfates and other organic sulfur forms, such as garlic, onion, and broccoli. Methionine and cysteine are both required for protein synthesis in the body, and diets must provide these two amino acids, or methionine alone, to meet optimal growth [32]. Some studies have highlighted their role in IBD and are discussed here, but further studies are needed to elucidate their direct role in the management and pathogenesis of IBD.

### 2.2. Nitrates, Nitrites, and Nitric Oxide

Sources of nitrates (NO_3_^−^) and nitrites (NO_2_^−^) include plant nutrients, additives to processed meats, and various dietary supplements. The FDA regulates allowable levels of inorganic nitrate and nitrate in water and foodstuffs, with the acceptable daily intake (ADI) of nitrate being 0–3.7 mg nitrate ion/kg body weight and the ADI of nitrite being 0–0.7 mg nitrite ion/kg body weight [33]. Common dietary sources of nitrates, which account for about 50–75% of the overall dietary intake of nitrates [34], include green leafy vegetables and root vegetables, including spinach and beets [35]. Nitrites are often used as preservatives in processed foods such as bacon, sausages, and deli meats, with an estimated 35–40% of dietary nitrite intake coming from these animal-based products, as well as other vegetables and fruits [34]. Some plant-based nitrites are generally associated with beneficial effects [36]. There are also beneficial cardiovascular effects associated with metabolites of nitrates, including a reduction in resting blood pressure and improved vascular function [35]. However, excessive consumption of nitrate and nitrite in processed foods can form potentially harmful N-nitroso compounds in the acidic environment of the stomach, especially in high quantities, which have been linked to an increased cancer risk. These effects are more pronounced compared to plant-derived nitrates, which typically exert protective effects [14]. Nitric oxide (NO) is an essential inorganic molecule with various different physiological and pathological implications. It is normally synthesized from the amino acid L-arginine via the enzyme nitric oxide synthase (NOS). However, inducible NOS (iNOS) is known to be highly expressed in inflammatory cells in response to immunogenic stimuli, and levels of NO are known to be increased in patients with IBD [37].

### 2.3. Ammonia and Urea

The gut microbiota is linked to nitrogen and ammonia metabolism, and changes to the gut microbiome can disturb this system [38]. Most of the ammonia in the gastrointestinal tract originates from protein catabolism, either from high-protein diets, deamination, or periods of starvation when muscle protein is catabolized to make energy. It is also naturally produced by gut flora [38,39]. Ammonia plays a role in various protein anabolic pathways, including the conversion of glutamate to glutamine. Glutamine that is transported from peripheral tissues to the liver can be broken down into ammonia and glutamate. From here, ammonia is incorporated into the hepatocyte mitochondria and ultimately forms urea [39]. Urea, a byproduct of this protein metabolism synthesized by the liver and excreted by the kidneys, can re-enter the gastrointestinal tract through salivary and gastric secretions. Via the enzyme urease, an enzymatic protein synthesized by bacteria, gut bacteria hydrolyze urea into ammonia, which can be utilized for microbial protein synthesis or contribute to local toxicity if overproduced. Mammals do not possess the urease gene, so the breakdown of urea in the colon is dependent only on gut microbiota [38]. This nitrogen recycling mechanism contributes indirectly to the nitrogen pool available for microbial processes, influencing microbial diversity and activity [40].

## 3. Dietary Nitrogen and Their Roles in IBD Pathogenesis

While there are a variety of colonic nitrogen sources (Figure 1), only a few have been studied, and their impact on gut permeability, mucus layers, and immunity is summarized here and in Table 1.

### 3.1. Effects on Gut Permeability

Glutamine, abundant in various food sources such as pork, fish, dairy, and eggs, is a vital substrate for the synthesis of proteins involved in maintaining the integrity of the intestinal barrier. Through activation of the mTOR signaling pathway, glutamine increases protein synthesis, promotes intestinal development, regulates tight junction protein expression, and inhibits apoptosis from oxidative stress [41,42]. It prevents hyperpermeability by stabilizing tight junctions in epithelial cells, which is critical in preventing microbial translocation and maintaining mucosal immunity [43,47]. Prior cellular studies have demonstrated that deprivation or inhibition of glutamine leads to decreases in intestinal epithelial cell barrier function through the reduction in various tight junction proteins [70,71]. Barrier function can, however, be rescued when glutamine is supplemented [70]. Prior studies in humans have also demonstrated that, in critically ill patients with bacteremia, sepsis, and multi-organ failure syndrome, there is an acute increase in intestinal permeability, and supplementation with glutamine reduces this permeability [72].

While arginine is also obtained from dietary sources, it can also be provided through body protein breakdown and endogenous production. Endogenously, arginine plays an important role in many metabolic pathways, including the production of urea, creatine, creatinine, nitric oxide, glutamine, and pyrimidine [72,73]. While healthy adults can obtain arginine from their diet or synthesize it themselves, IBD patients have an altered absorptive capacity of the gut and often cannot obtain enough arginine through their diet. The uptake of arginine at the cellular level depends on cationic amino acid transporters (CAT). Of the various isoforms of this protein, CAT1 is directly involved in the uptake of arginine, and CAT2 (also known as SLC7A2) is an inducible form of the transporter [73]. Animal studies have shown that SLC7A2, an arginine transporter, plays the largest role in IBD pathogenesis, and mice lacking this protein are shown to be more susceptible to dextran sulfate-induced colitis [74]. IBD patients are known to have decreased expression of SLC7A2 in the colonic mucosa, with a subsequent decrease in levels of arginine in colonic tissue, suggesting a direct role of this protein in the development of mucosal injury in IBD patients [75,76]. Animal studies have also demonstrated that mice with experimentally induced colitis had higher levels of intestinal permeability and bacterial translocation, and rats consuming arginine-supplemented diets showed a decrease in this intestinal permeability and bacterial translocation, likely secondary to reduced inflammation and improved mucosal architecture [77].

Arginine is also a precursor for NO production. Reactive nitrogen species (RNS) include NO, which is produced by nitric oxide synthase (NOS) through consumption of arginine and citrulline. Prior studies have shown that NO production is significantly increased in inflamed colonic tissue of IBD patients due to increased activity of inducible nitric oxide synthase (iNOS) [54]. Increased iNOS expression is associated with epithelial cells and inflammatory infiltrates in the mucosa of the colon, especially during states of inflammation [55]. Earlier studies have shown a close relationship between the upregulation of iNOS in intestinal epithelial cells and the initiation and maintenance of inflammation in IBD [54]. NO produced during these states can further produce reactive nitrogen species that can interact with certain cellular components, leading to DNA damage and disruption of protein function, as well as impaired cellular mechanisms and worsening chronic inflammation in IBD [78,79]. However, while NO has been shown to exacerbate inflammation in the gut and alter barrier function, there are some protective effects of NO in IBD pathogenesis as well. Through enhancing the expression of peroxisome proliferator-activated receptor (PPAR), NO can provide some alleviation of the inflammatory response seen in IBD. However, studies have demonstrated that inhibition of iNOS displays a protective effect in IBD through shunting of arginine into other metabolic pathways that are beneficial for gut health [80]. Thus, while the true effector function of NO in IBD is complex and not completely understood, there is ultimately a benefit in reducing NO in the gut [56].

### 3.2. Effect of Nitrogen on Impaired Mucus Layers → Exposure to Nitrogen

The mucosal layer of the GI tract provides adequate defense against pathogens and helps contain various microorganisms and molecules in the gut [81]. Alterations in this mucosal barrier play a role in the pathogenesis of IBD. One important factor in the development of IBD is the loss of homeostasis in response to pathogens and commensal organisms in the gut, resulting in an altered immune response. The mucosal layer and its homeostasis are directly involved in the mechanism [82,83]. In addition to bacteria and various metabolites, dietary compounds can alter the mucosal layer in IBD patients. Dietary components, including complex proteins, are ingested and further hydrolyzed in the GI tract, releasing food-derived peptides and free amino acids [84]. Various studies have demonstrated that total proteins, protein hydrolysates, and bioactive peptides from various dietary sources can modulate levels of mucus, ultimately altering the barrier protecting against IBD [81].

Inflammation in the gut mucosa is also associated with changes in tissue metabolism, depletion of local nutrients, imbalances in oxygen supply and demand, and increased production of reactive oxygen (ROS) and nitrogen species (RNS) [85,86]. As previously described, free radicals can be released in IBD patients [87]. Increased activity of neutrophils and macrophages seen in chronic inflammatory states, such as IBD, can increase levels of ROS or RNS, which have been shown to be correlated with the severity of inflammation in the mucosal layer of the colon [88]. Excess NO can damage the intestinal mucosa and the epithelial barrier, leading to inflammation and tissue damage [89,90]. Interestingly, in mice that were induced with intestinal inflammation, treatment with nano antioxidant particles, which are zinc ions combined with tannic acids, has anti-inflammatory properties and can scavenge free radicals, removing a variety of ROS and RNS and preventing intestinal cellular damage mediated by these particles. They also promoted the healing of intestinal mucosa, suggesting the direct role of NO and various other ROS/RNS in IBD pathogenesis [86].

Another example of the role of reactive species is through the formation of peroxynitrite (ONOO-), which is a reactive oxidant formed by the reaction of NO with O_2_-. ONOO- can induce damage to cellular structures, including mucosal membranes, and exacerbate inflammation and tissue injury [54]. Mucosal damage seen in IBD patients further allows for excessive translocation of luminal antigens and bacteria, which are another key driver of inflammation in IBD [91].

Arginine, a key regulator of intestinal barrier function, also plays a role in mucosal integrity. Arginine is converted to ornithine via the enzyme arginase. Ornithine is used by ornithine decarboxylase to produce the polyamine putrescine, which is further metabolized to spermidine and spermine. The compounds are polyamines that are directly associated with mucosal protection in the GI tract [44]. Additionally, oral arginine supplementation in animal models has been shown to attenuate degrees of intestinal damage seen in intestinal ischemia, leading to the healing of gut mucosa [45]. In other animal models of experimentally induced colitis, dietary supplementation with arginine dampened pro-inflammatory responses and inflammatory cell infiltration, leading to improved mucosal integrity [46].

Hydrogen sulfide (H_2_S), a byproduct of bacterial metabolism of sulfur-containing compounds like cysteine and sulfate, plays a complex role in gut physiology. At lower concentrations, H_2_S can actually support epithelial health—acting as a signaling molecule that promotes mitochondrial respiration, aids in tissue repair, and supports mucus production [92,93]. However, at higher luminal levels, H_2_S shifts from being protective to harmful. It has been shown to inhibit cytochrome c oxidase in colonocyte mitochondria, reduce ATP production [19], impair butyrate metabolism, and increase oxidative stress—ultimately weakening the epithelial barrier and slowing cell renewal [94,95]. This dose-dependent effect reflects the dual nature of H_2_S: beneficial in small amounts, but disruptive when elevated. Recent microbiome studies suggest that cysteine-fermenting species such as *Clostridium*, *Fusobacterium*, and *Desulfovibrio* may be prominent contributors to colonic H_2_S pools, sometimes more so than classical sulfate-reducing bacteria [96]. In the context of IBD, where mucosal redox balance and energy metabolism are already impaired, excess microbial H_2_S may further strain epithelial defenses and contribute to chronic inflammation.

Ammonia, a byproduct of bacterial amino acid deamination and urea metabolism, has been shown to alter colonocyte energy metabolism in a concentration-dependent manner. In a study using isolated rat colonocytes, Cremin et al. demonstrated that ammonia significantly inhibited the oxidation of butyrate and acetate—two primary energy sources for colonic epithelial cells—by reducing oxygen consumption rates. Interestingly, they found that this inhibitory effect could be partially reversed with glucose supplementation, suggesting a shift in substrate utilization under ammonia stress [97]. In a separate in vivo study, Andriamihaja et al. (2010) showed that high-protein diets in rats led to elevated intraluminal ammonia, which was associated with impaired mitochondrial respiration, reduced colonic ATP content, and structural disruption of colonocyte membranes [98]. These metabolic impairments are particularly relevant in the context of IBD, where reduced SCFA oxidation and mitochondrial dysfunction may contribute to barrier breakdown and ongoing mucosal inflammation.

Certain aromatic amino acid–derived bacterial compounds also contribute to epithelial dysfunction and immune activation in the gut. One such compound, *p*-cresol, is produced through bacterial fermentation of tyrosine and can accumulate at millimolar concentrations in the colon. *p*-Cresol has been shown to impair mitochondrial oxygen consumption in colonocytes, leading to reduced ATP production, increased oxidative stress, and disrupted epithelial barrier integrity [19]. These effects are accompanied by decreased epithelial proliferation and increased paracellular permeability—changes that may contribute to chronic mucosal inflammation observed in IBD. Additional studies have demonstrated that *p*-cresol not only disrupts colonocyte energy metabolism but also increases superoxide production and exhibits genotoxic properties, especially at higher luminal concentrations [99]. In murine models, *p*-cresol has also been shown to suppress gut hormone expression, such as GLP-1, and accelerate small intestinal transit, further altering mucosal exposure and nutrient signaling pathways [100]. Genotoxicity assays using physiologic doses of *p*-cresol have confirmed its ability to damage colorectal epithelial DNA and impair repair mechanisms [101]. Together, these findings underscore the multifaceted impact of *p*-cresol—ranging from cellular metabolism and barrier dysfunction to systemic hormone regulation—highlighting its relevance as a nitrogen-derived microbial metabolite in the pathogenesis of IBD.

While excess bacterial metabolites such as H_2_S and ammonia can disrupt colonocyte function, intestinal epithelial cells have evolved detoxification mechanisms to manage these challenges. For hydrogen sulfide, colonocytes primarily rely on mitochondrial enzymes such as sulfide:quinone oxidoreductase (SQR), followed by thiosulfate sulfurtransferase (TST) and ETHE1, to convert H_2_S into less reactive sulfur compounds. This enzymatic pathway is especially active in differentiated epithelial cells and plays a key role in preventing H_2_S-induced mitochondrial inhibition while allowing its use as a respiratory substrate at low concentrations [102]. Ammonia detoxification, though better characterized in hepatic tissue, also occurs in the intestinal epithelium. Colonocytes express glutamine synthetase (GS), which facilitates the incorporation of free ammonia into glutamine—a process that not only prevents toxic ammonia buildup but also supports nitrogen balance and cellular metabolism. Extrahepatic GS expression has been observed in various epithelial tissues, including the gut, and may serve as an adaptive mechanism to buffer excess ammonia locally [103]. These detoxification processes are essential to maintaining epithelial homeostasis and may become overwhelmed in inflammatory conditions such as IBD.

Multiple studies now suggest that the ability of the intestinal epithelium to detoxify H_2_S is compromised in IBD patients. For example, quantitative analysis of colonic biopsies from ulcerative colitis (UC) patients revealed significantly reduced activity and gene expression of thiosulfate sulfurtransferase (TST), a key enzyme in the H_2_S-oxidation pathway, compared to controls [104]. In related work, immunohistochemical analysis showed lower levels of other detoxifying enzymes—such as SQOR and ETHE1—in both Crohn’s and UC tissue, indicating a general decline in epithelial sulfide metabolism across IBD subtypes [92]. The consequence of this enzymatic shortfall is twofold: it can lead to unchecked H_2_S accumulation, which inhibits mitochondrial respiration, and it may impair butyrate oxidation—both contributing to epithelial energy deficits and barrier dysfunction. This reduced detoxification capacity likely exacerbates mucosal injury and sustains inflammation in IBD.

### 3.3. Gut Immune System

Recent studies suggest that nitrogen-containing dietary compounds may play a role in modulating mucosal immunity through effects on B-cell function and IgA production. Amino acids and their bacterial metabolites can enhance intestinal IgA responses, potentially strengthening mucosal defense mechanisms [105]. Additionally, gut microbial shifts driven by nitrogen availability may influence B-cell class switching to IgA within Peyer’s patches and isolated lymphoid follicles via mechanisms involving B-cell activating factor (BAFF) and a proliferation-inducing ligand [106]. These pathways function independently of T cells and are critical for maintaining gut immune balance. Secretory IgA, in turn, can selectively coat nitrogen-metabolizing bacterial taxa, contributing to the regulation of microbial composition and dampening immune activation [107]. Together, these findings support a bidirectional relationship between nitrogen metabolism and mucosal immunity that may be particularly relevant in the context of IBD.

Glutamine supplementation in humans has also been shown to improve secretory IgA production, which plays a crucial role in neutralizing pathogens at the gut mucosal surface [42]. Additionally, glutamine supports the proliferation and activation of immune cells such as lymphocytes and macrophages while also enhancing cytokine production and modulating inflammatory responses, ensuring balanced immune activity in the gut [108]. It further enhances the production of IL-10, an anti-inflammatory cytokine critical for maintaining mucosal immunity. IL-10 suppresses the secretion of pro-inflammatory cytokines such as TNF-α, IL-1β, and IL-6 by modulating macrophage and T-cell activity. Studies have shown that glutamine supplementation increases IL-10 levels in IBD models, promoting an anti-inflammatory environment and aiding in mucosal healing [48]. Glutamine also influences the NF-κB and PI3K-Akt pathways, reducing pro-inflammatory signaling while supporting tissue repair and immune regulation [43]. Through indirectly reducing IL-6 levels, a known pro-inflammatory cytokine, glutamine improves epithelial barrier function and decreases microbial translocation, which are known to be triggers for IL-6 secretion in the gut. High IL-6 levels are associated with worsened disease activity in IBD and are known therapeutic targets in the treatment of IBD [109]. As previously discussed, a consistent finding in experimental animal and human IBD studies is the upregulation of iNOS and increased production of NO. Elevated levels of NO in both serum and affected tissues of IBD patients are mainly synthesized by iNOS and can exacerbate GI inflammation [37].

Further, in vitro inflammatory models of IBD with insufficient levels of glutamine supply lead to increased sensitivity of colonic epithelial cells to cytokine-induced cell death, and sufficient glutamine supplementation in both dietary in vivo models and in vitro models was essential for colonic epithelial cells to mount resistance to cell death induced by inflammation, suggesting a role for dietary glutamine supplementation in IBD [110].

Methionine supplementation has also been demonstrated to have protective effects on the gut through various pathways in experimental IBD models. The activation of the NRF2 pathway, which plays an important role in oxidative stress, was shown to be downregulated in IBD-induced animal models, and dietary supplementation with D-methionine reversed this effect, suggesting a possible anti-inflammatory role of methionine in IBD [49]. Mice receiving dietary D-methionine supplementation in these studies also exemplified histological disease improvement in the colon and showed protective effects on the gut injury seen in these IBD models. Methionine deficiency in animal models with experimentally induced colitis has further been shown to weaken immune cell adhesion and increase exposure between intestinal bacteria and immune cells. However, there were also increases in anti-inflammatory cytokines in these studies, suggesting methionine deficiency in IBD could promote regeneration of immune regulatory functions [50]. Liu et al. further demonstrated this concept. Mice with experimentally induced colitis that were given methionine-restricted diets showed significant suppression of NF-kB activation, consistent with those in human patients with IBD and suggesting that methionine restriction could be used as an immunomodulator and anti-inflammatory modulator in IBD patients [51]. Methionine is, therefore, a potential factor in modulating the immune system in IBD patients; however, further human studies are needed to differentiate its role in managing patients.

Although both Crohn’s disease (CD) and ulcerative colitis (UC) are associated with microbial dysbiosis and altered nitrogen metabolism, emerging data suggest that the mechanisms involved may differ between the two conditions. In CD, studies have reported elevated fecal concentrations of amino acids and ammonia, which may be driven by increased bacterial urease activity and a higher abundance of nitrogen-metabolizing taxa, particularly within the Proteobacteria phylum, such as *Escherichia coli* and *Klebsiella* species [18]. These taxa have been implicated in sustaining inflammation and promoting dysbiosis in CD. In contrast, UC appears to be characterized more prominently by impaired epithelial barrier function and mucus layer disruption, which may increase exposure of the colonic mucosa to nitrogen-derived microbial metabolites and contribute to immune activation [111,112]. Metagenomic analyses further indicate that microbial communities in CD are enriched for genes involved in nitrogen respiration pathways, while UC samples tend to show increased expression of genes associated with oxidative stress and tight junction impairment [113]. These findings support the idea that while both diseases involve nitrogen dysregulation, the dominant pathways and microbial contributions may be distinct, with CD more tightly linked to microbial nitrogen metabolism and UC more influenced by epithelial barrier dysfunction.

## 4. Effects of Nitrogen-Containing Compounds on Gut Microbiota and Their Roles in IBD

The majority of dietary protein is absorbed in the small intestine (>90%), but the fraction that remains reaches the colon and serves as a substrate for microbial metabolism [22,23]. The overall amounts of protein reaching the colon also directly correlate with the amount of protein consumed in the diet. Unabsorbed proteins, peptides, and carbohydrates that reach the colon undergo putrefaction and fermentation by gut microbes, producing metabolites such as ammonia, short-chain fatty acids (SCFAs), and hydrogen sulfide, which can impact gut health and inflammation [16]. These bacterial metabolites have diverse effects on the host.

Nitrogen products in the gut have been shown to decrease in a longitudinal gradient from the proximal small intestine to the large intestine, mostly due to host absorption of dietary nitrogen [114]. In mice studies, the availability of this nitrogen in the colon is from both dietary and host-secreted pathways, with no significant difference between pathways. Members of the *Bacteroidales* order have been shown to consume higher amounts of this nitrogen more readily than other bacterial taxa in the colon [114]. Igai et al. have further demonstrated nitrogen fixation activity in the human gut microbiome, which corresponded to about 0.01% of human nitrogen requirements, suggesting a potential contribution of gut microbiota to host nitrogen metabolism [12]. As previously discussed, bacteria in the gut survive on carbohydrates and nitrogen. In prior mice studies of various different diets comprising different amounts of protein, carbohydrates, and fats, the abundance of microbial species in the gut varied in response to the ratios of protein and carbohydrate intake [115]. While the known positive interactions of the gut microbiome are supported by high-carbohydrate diets, these benefits are relative to the protein intake of the host animal [115], and the availability of intestinal nitrogen to microbes in the large intestine plays a vital role in regulating the interactions between gut microbes and their host.

Dietary nitrogen interacts with the gut microbiota primarily in the colon. Here, it plays an important role in microbial growth and metabolism, and these processes depend on an adequate nitrogen supply, which, when limited, affects their growth and abundance [114,115]. The colon experiences nitrogen limitation due to upstream host absorption of dietary nutrients in the small intestine [114]. Microbial community assembly in the gut is fundamentally shaped by bacterial strategies to access nitrogen [115]. Host-secreted amino acids contribute significantly to the nitrogen available in the colon, accounting for an appreciable fraction of nitrogen accessible to gut bacteria [114]. The protein-to-carbohydrate (P:C) ratio in the diet strongly affects the bacterial community response, as carbohydrates contain no nitrogen while protein does. Vegan and vegetarian diets are associated with a higher abundance of microbial genes and proteins responsible for the synthesis of essential amino acids, indicating adaptation to lower dietary protein intake [116]. Increased nitrogen availability from dietary protein can lead to changes in microbial metabolism and potentially impact host health [22]. In a large retrospective cohort study examining dietary carbohydrate intake and mortality, the P:C ratio was shown to correlate with mortality in humans, with higher rates of mortality seen in diets with either <40% or >70% of energy from carbohydrates, and lower rates of mortality seen with 50–55% of energy from carbohydrates. Low carbohydrate diets that replaced energy from carbohydrates with energy from animal-derived protein or fat were also associated with greater risk. However, when energy from carbs was replaced with plant-derived protein or fat, this association was reversed [117]. These sources highlight the importance of dietary nitrogen, particularly from protein, in shaping the gut microbiome and its interactions with the host in the colon.

The balance of bacteria in the gut plays a key role in overall immune regulation and digestive health, and gut microbiota dysbiosis is a key pathway of disease in IBD. Prior metagenomic and metabolomic studies in patients with IBD have demonstrated an association between disease severity, gut dysbiosis, and bacterial production of free amino acids [18]. For example, studies have also shown an increase in the Proteobacteria phylum in IBD, leading to intestinal inflammation [95,96,97]. These bacteria exhibit increased expression of urease-utilizing gene pathways, including those involved in nitrogen metabolism, biosynthetic pathways, and sulfur relay systems. This increased utilization of urease promotes dysbiotic microbial growth [18]. Increased levels of ammonia and hydrogen sulfide in the gut lumen have further been implicated in exacerbating inflammation and tissue damage in IBD [59]. As a result, urease-mediated nitrogen influx into the gut microbiota may play a crucial role in the dysbiosis observed in IBD patients.

As previously discussed, patients with IBD are known to have increased levels of NO in the gut. However, the role of NO on the gut microbiota and the relationship between NO, gut bacteria, and IBD have not been widely studied. Leclerc et al. performed an in vitro study with human fecal samples to understand the effects of NO on microbiota. They showed that high NO concentrations depleted the microbiota of beneficial butyrate-producing species and led to an increased shift in the production of potentially deleterious species, such as *E. coli*, *E. faecalis*, and *P. mirabilis* [118], ultimately suggesting that NO can participate in the cycle of inflammation seen in the gut during certain disease states.

The proteolytic activity in the colon is largely performed by certain bacteria, including those in the following genera: *Bacteroides*, *Clostridium*, *Propionibacterium*, *Fusobacterium*, *Streptococcus*, and *Lactobacillus* [119]. Prior studies have demonstrated the involvement of microbial proteases in IBD, particularly bacteria such as *Staphylococcus aureus*, *Clostridium difficile*, *Clostridium perfringens*, *enterohaemorrhagic E.coli*, *enterotoxigenic E. coli*, *Helicobacter pylori*, and *Bacteroides fragilis* [120,121].

Gut bacteria play a crucial role in nitrogen cycling through processes such as ammonification and nitrification. Dietary nitrogen compounds, including proteins, are metabolized by colonic bacteria to release ammonia, hydrogen sulfide, and other nitrogenous metabolites. High-nitrogen diets, particularly those rich in animal proteins, alter the gut microbiota’s composition by promoting the growth of proteolytic bacteria such as Bacteroides and Clostridia. These shifts can increase the production of harmful metabolites like ammonia and hydrogen sulfide while reducing beneficial microbes like SCFA producers [122]. Ammonification involves the breakdown of amino acids into ammonia, which can be further utilized by bacteria for microbial protein synthesis or excreted into the gut lumen [16]. Nitrification, though less prominent in the gut, facilitates the conversion of ammonia to nitrate by specialized bacteria under certain conditions [17].

Nitrates (NO_3_^−^) and nitrites (NO_2_^−^), commonly derived from dietary sources, significantly influence gut microbiota composition and metabolism. Their dual effects on microbiota and host physiology have implications for inflammatory bowel disease (IBD) pathogenesis. In healthy humans, dietary nitrate is well-absorbed in the upper gastrointestinal tract, but a considerable amount of the daily nitrate intake (about 33%) has been shown to reach the lower intestine [123]. Once ingested in the diet, nitrates are reduced to nitrites by oral and gut bacteria and further metabolized by gut bacteria into nitric oxide, which has vasodilatory and anti-inflammatory properties and has roles in vascular health and microbiota modulation [53]. Alternatively, nitrates in the inflamed gut act as electron acceptors for facultative anaerobes like *Enterobacteriaceae*, promoting growth and the production of harmful metabolites such as reactive oxygen species (ROS) [57]. Nitrate respiration also favors nitrate-reducing bacteria such as *Proteobacteria*, disrupting the balance of commensal anaerobes, including SCFA-producing microbes. This shift reduces beneficial SCFAs like butyrate, which is crucial for gut barrier integrity and anti-inflammatory signaling [58].

**Figure 2:**
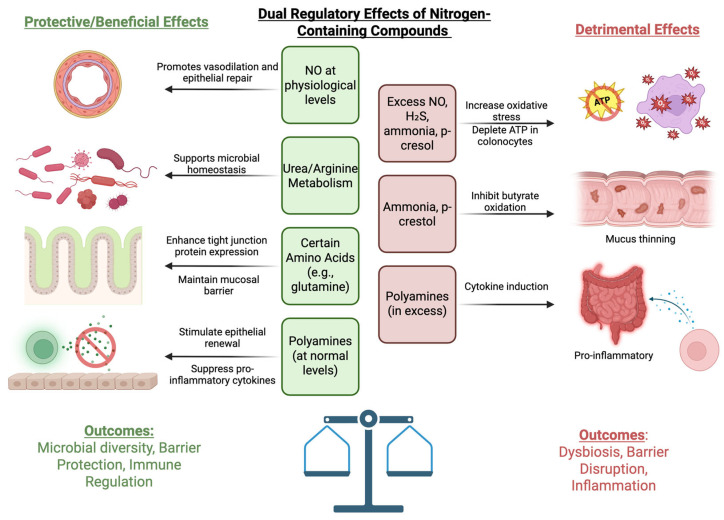
The positive and negative effects of gut microbial-derived metabolites from dietary nitrogen sources on the colon, including their physiological and biochemical impacts. Figure created using Biorender.

With respect to the gut microbiota, glutamine has been shown to alter microbial composition through its role in increasing beneficial microbes and decreasing microbes that are associated with dysbiosis, such as *Enterobacteriaceae*. These alterations can affect gut health and reduce inflammation by enhancing the production of anti-inflammatory metabolites [124]. In inflammatory bowel disease (IBD), glutamine supplementation has been associated with reduced disease severity in some preclinical studies [125]. Other studies have demonstrated that glutamine supplementation reduces intestinal permeability and enhances IL-10 production, leading to improved outcomes in patients with Crohn’s disease [43]. However, differing studies report minimal effects of glutamine on Crohn’s disease activity indices, suggesting variability in patient response based on microbiota composition and disease severity. Concerning arginine, mouse models of experimentally induced colitis fed diets supplemented with high levels of arginine showed an increase in the diversity of the intestinal microbiota. Arginine specifically leads to an increased abundance of the Bacteroidetes phylum, bacteria that are known to be decreased in IBD patients, suggesting a possible protective effect of arginine supplementation through the restoration of gut bacterial diversity [126]. A summary of the effects of gut microbial-derived metabolites on the colon as discussed previously can be found in Figure 2.

## 5. Other Notable Compounds and Their Effects on the Gut Microbiota and IBD

### 5.1. Lectins

Lectins, nitrogen-rich proteins found in many fruits, vegetables, legumes, and whole grains, contribute to the nitrogen pool through their role in plant defense mechanisms. While lectins are less digestible compared to other proteins, they may modulate gut microbiota composition by providing substrates for fermentation. Legumes, such as lentils and beans, are particularly rich in lectins, making them a significant contributor to dietary nitrogen intake, though their effects on gut health depend on preparation and cooking methods to reduce potential toxicity [28]. Lectins are increasingly recognized for their potential prebiotic effects and influence on gut microbiota composition. In the body, they primarily serve as recognition molecules and mediate cell-to-cell and cell-to-molecule interactions [60]. The role of lectins is diverse, and includes infection defense, innate immunity, glycoprotein synthesis, and cell cycle regulation.

In rodent models, specific lectins like Phytohemagglutinin lectin (PHA) have been shown to reduce the mucus lining of the gut, allowing local microbes like *E. coli* and *Streptococcus* species to increase in colonized amounts [61]. Other studies have demonstrated increased intestinal permeability, abnormal microvilli structure, and changes in acid secretion and nutrient absorption in rats [62,127,128]. While several works have highlighted the roles of dietary lectins in relation to inflammation and autoimmunity, including increases in intestinal permeability, very little has been studied with respect to lectin-induced pathogenesis in IBD [129]. Konozy et al. have summarized the various effects of dietary lectins on the immune system, including increased intestinal wall leakiness, as well as both pro- and anti-inflammatory properties [60]. Despite receiving attention from scientists due to the potential role of dietary lectins in initiating autoimmune diseases, the exact link to pathogenesis remains unknown. More recent studies of human commensal bacterium encoded lectin have demonstrated an interaction of this bacterial lectin with myeloid cells in the blood and intestine, possibly suggesting a role of lectins in mediated interactions between commensal bacteria and the human host [63]. However, further studies are needed to better understand the exact role of lectins in autoimmunity and IBD.

### 5.2. Purines

Recent research has shown that gut bacterial metabolism contributes to host purine homeostasis, which can have implications for inflammatory processes in the intestine. During times of injury, infection, and wound healing, the intestinal mucosa requires high levels of nucleotides for energy, cell proliferation, and immune function. In murine models, the gut microbiota has been shown to be a source of purines that are available to the intestinal mucosa for use. In colonic tissue lacking purine-producing microbiota, detrimental effects on the gut mucosal barrier were observed, and the proliferation of intestinal epithelial cells was overall stunted [64]. Further studies exploring expressions of genes related to purine metabolism have shown increases in expression of purine metabolism genes in patients with IBD, implicating a role for purine metabolism in the immune-related pathways of IBD [65]. Additionally, in vivo and in vitro studies have demonstrated disrupted cell proliferation, which attenuates wound healing and increases tissue damage, in experimental colitis models treated with allopurinol, an inhibitor of purine salvage [66]. A defective mucus layer with increased contact between microbiota and mucosa is a key hallmark of IBD pathogenesis [67,130], suggesting that augmenting gut purine availability in IBD patients could provide a benefit to the gut mucosal barrier. Additionally, recent genetic studies have highlighted purine metabolism genes closely related to IBD. The gut microbiota is known to metabolize purines to uric acid, and high-purine diets, such as those containing seafood and alcohol, have also been linked to changes in the gut microbiota composition in animal models. These changes are associated with increased levels of uric acid and an increased risk of gout. About 20% of uric acid in the body is derived from exogenous purines, and higher levels are a feature of gout [68]. Interestingly, IBD is strongly associated with gout, suggesting a connection between purine metabolism and IBD [69].

## 6. Potential Therapeutic Implications

Excessive nitrogen intake, particularly from protein sources, promotes the production of harmful metabolites such as ammonia and hydrogen sulfide, which are linked to epithelial damage and inflammation. Modulating nitrogen intake by reducing protein consumption or shifting to plant-based protein sources can help lower the production of these metabolites, potentially alleviating IBD symptoms [131].

Certain nitrogenous compounds have demonstrated anti-inflammatory effects. Amino acids such as glutamine and arginine are crucial for maintaining mucosal integrity and reducing inflammation. Glutamine supplementation, for instance, supports the repair of damaged epithelial cells, while arginine has been shown to enhance immune regulation and nitric oxide synthesis, contributing to reduced IBD activity [52].

Personalized dietary regimens tailored to an individual’s microbiome and metabolic profile are gaining traction in the management of IBD. By identifying specific dietary triggers and beneficial nitrogen sources, interventions can target dysbiosis and inflammation more effectively. Such approaches may involve increasing the intake of plant-based proteins, prebiotics, and amino acids, either through supplemental or dietary methods, to support a balanced microbiota and anti-inflammatory metabolite production [132]. While the gut microbiota influences dietary metabolism and immune responses, its composition varies significantly among individuals. Personalized dietary interventions utilize baseline microbiota profiles to predict dietary responses and tailor nutritional strategies. For example, studies have shown that individuals with higher levels of butyrate-producing bacteria respond better to prebiotic and fiber-rich diets, which support anti-inflammatory pathways [133]. Predictive analytics have also been applied to understand diet-microbiome interactions. For example, computational models assessing changes in specific bacterial taxa (e.g., *Clostridia* and *Bacteroides*) linked to dietary interventions such as the IBD-Anti-Inflammatory Diet (IBD-AID) have also informed personalized nutritional therapies for improving microbial diversity and reducing inflammation [134].

Studies highlight the potential of dietary interventions that limit nitrogen-rich compounds linked to harmful metabolites while enhancing nitrogen sources that promote gut health. The Mediterranean diet, characterized by its low nitrogen load from red meat and high levels of plant-based proteins and fibers, has shown promise in reducing inflammation and improving gut microbiome composition in IBD patients [135]. The Mediterranean Diet, which consists of a total protein intake that is about 20% lower and animal protein intake that is 50 to 60% lower compared to typical Western diets [136], has shown promise in reducing inflammation and improving symptoms of IBD. Studies have shown that these interventions can also increase beneficial microbial taxa while reducing harmful metabolites [135]. For example, healthy patients consuming higher levels of dietary components consistent with those in the Mediterranean diet, such as increased content of plant-based food, have been shown to have beneficial changes in microbiome-related metabolic profiles [137]. Other benefits of the Mediterranean diet that may be relevant to IBD pathogenesis include increased protection against oxidative stress and inflammation, as well as increased gut microbiota-mediated production of metabolites that positively affect host function [136].

In IBD patients specifically, Haskey et al. have demonstrated that IBD patients consuming a Mediterranean diet pattern showed induced alterations in the gut microbiome that are consistent with those seen in clinical remission. There were also induced changes in the microbial species associated with a protective role in IBD, as well as the production of short-chain fatty acids [138]. While the beneficial effects of the Mediterranean diet and its high content of plant-based proteins have been demonstrated in various populations, and the overall benefits of these diets have been shown in IBD patients, further studies are needed to explain how the nutrient composition can be adjusted to better suit patient needs. Additionally, dysbiosis in IBD patients often involves an overgrowth of pro-inflammatory taxa such as *Proteobacteria* and reduced levels of beneficial bacteria like *Firmicutes*, and personalized diets aim to correct this imbalance using targeted prebiotics, probiotics, or dietary exclusions [139]. However, personalized nutrition for IBD patients faces barriers such as the heterogeneity of microbiome-disease interactions and the lack of standardized clinical guidelines. Additionally, patient adherence and the cost of microbiome testing remain significant challenges [132].

## 7. Discussion

This review highlights the multifaceted role of dietary nitrogen compounds in the pathogenesis and management of inflammatory bowel disease (IBD), with a specific focus on their interactions with the gut microbiome and immune system. While advancements in sequencing technologies have provided insights into gut microbiota composition and function, the complex interplay between dietary nitrogen sources, microbial metabolism, and IBD pathophysiology remains poorly understood.

A key finding from this review is the significant influence of dietary amino acids, nitrates, and urea on gut microbial ecology and immune modulation. Amino acids such as glutamine and arginine, derived from dietary proteins, are essential for maintaining gut barrier integrity and modulating inflammation. Glutamine, for instance, enhances tight junction protein expression, reduces intestinal permeability, and promotes anti-inflammatory cytokine production, such as IL-10. Similarly, arginine supports mucosal healing and immune regulation, though its metabolism into nitric oxide (NO) via inducible nitric oxide synthase (iNOS) can exacerbate inflammation under dysregulated conditions. These findings underscore the duality of nitrogen metabolism in IBD, where certain compounds exhibit both protective and deleterious effects depending on their concentrations and metabolic pathways.

Nitrates and nitrites, often associated with plant-based diets, have demonstrated beneficial cardiovascular and anti-inflammatory properties, primarily through their conversion to NO. However, in the inflamed gut environment characteristic of IBD, these compounds can serve as electron acceptors for facultative anaerobes like *Proteobacteria*, promoting microbial dysbiosis and the production of harmful metabolites such as reactive oxygen species (ROS). This dynamic highlights the need for a nuanced understanding of dietary nitrate sources, as plant-derived nitrates may provide protective effects, whereas processed food-derived nitrates may exacerbate inflammation.

Another critical aspect of nitrogen metabolism in IBD is the production of ammonia and other nitrogenous metabolites by gut microbes. Ammonia, derived from protein putrefaction and urea hydrolysis, has been implicated in epithelial barrier dysfunction and inflammatory responses. Increased proteolytic activity in the colon, driven by dysbiotic bacteria such as *Bacteroides* and *Clostridia*, further exacerbates this inflammatory milieu. Interventions targeting ammonia production, such as dietary modulation or the use of urease inhibitors, hold potential as therapeutic strategies for reducing inflammation and improving gut health.

The interplay between dietary nitrogen compounds and gut microbiota extends beyond direct metabolic interactions. For instance, the reduction in beneficial short-chain fatty acid (SCFA)-producing bacteria, commonly observed in IBD, may be indirectly influenced by nitrogen availability. While SCFAs like butyrate are primarily produced through carbohydrate fermentation, nitrogen-containing compounds help maintain microbial nitrogen balance, enabling optimal carbohydrate fermentation. This suggests that dietary interventions promoting both SCFA production and nitrogen balance could synergistically enhance gut health and reduce inflammation.

Despite these advancements, significant gaps remain in our understanding of nitrogen metabolism in IBD. The heterogeneity of dietary nitrogen sources, coupled with individual variations in gut microbiota composition and host immune responses, presents challenges in developing universally effective interventions. Personalized nutrition approaches, informed by microbiome profiling and metabolic analyses, offer promising avenues for tailoring dietary strategies to individual needs and preferences. For example, plant-based diets rich in fiber and low in animal proteins have shown potential in reducing inflammation and improving microbiome diversity in IBD patients. However, these strategies require further validation in clinical settings to establish standardized guidelines.

In conclusion, this review highlights the complex relationship between dietary nitrogen compounds, gut microbiota, and the pathophysiology of IBD. While nitrogen metabolism represents a promising target for therapeutic intervention, further research is needed to elucidate the precise mechanisms by which dietary nitrogen impacts gut health. Future studies should prioritize integrative approaches that combine microbiome profiling, metabolic analyses, and clinical outcomes to develop personalized dietary strategies for managing IBD.

## Figures and Tables

**Table 1:** Summary of dietary nitrogen sources and their effects on gut health and IBD.

Nitrogen Source	Primary Source	Microbial Metabolites	Effects on Gut Barrier	Effects on Inflammation	IBD-Specific Notes
Amino Acids (e.g., Glutamine, Arginine, Methionine)	Meat, fish, dairy, legumes, soy	SCFAs, ammonia, polyamines	↑ Tight junction integrity (via mTOR, CAT1/2); ↑ mucus production [41,42,43,44,45,46]	↓ Pro-inflammatory cytokines (e.g., IL-6); ↑ IL-10 [42,47]	Glutamine and arginine support mucosal repair; methionine has mixed effects [48,49,50,51,52]
Nitrates/Nitrites	Leafy greens, beets, cured meats	Nitric oxide (NO), N-nitroso compounds	Plant-based nitrates may preserve barrier; processed forms can ↑ permeability [35,36,53]	NO has dual roles: anti-inflammatory via PPAR-γ or damaging via iNOS [54,55,56]	NO production ↑ in IBD; excess favors dysbiosis and oxidative damage [54,57,58]
Urea/Ammonia	Endogenous metabolism, protein catabolism	Ammonia	↑ Permeability when overproduced; toxic to epithelium [38,39]	↑ Inflammatory cytokines, oxidative stress [18,59]	↑ Urease-expressing bacteria in IBD; ammonia worsens barrier function [18,38,40]
Lectins	Beans, lentils, whole grains	—	Rodent studies: ↓ mucus thickness, ↑ epithelial permeability [60,61,62]	Mixed: Some prebiotic effects; others increase ROS & permeability [60,61]	Human studies limited; may alter barrier and immune signaling in IBD [60,63]
Purines	Seafood, organ meats, beer	Uric acid	↓ Barrier function if depleted; required for cell proliferation [64,65]	Excess linked to inflammation and immune dysregulation [65,66,67]	High-purine diets → dysbiosis, ↑ IBD-gout comorbidity [68,69]

↑ increasing; ↓ decreasing; → can promote.

## References

[1] Qiu P., Ishimoto T., Fu L., Zhang J., Zhang Z., Liu Y. (2022). The Gut Microbiota in Inflammatory Bowel Disease. Front. Cell. Infect. Microbiol..

[2] Zheng J., Sun Q., Zhang J., Ng S.C. (2022). The role of gut microbiome in inflammatory bowel disease diagnosis and prognosis. United Eur. Gastroenterol. J..

[3] Khan I., Ullah N., Zha L., Bai Y., Khan A., Zhao T., Che T., Zhang C. (2019). Alteration of Gut Microbiota in Inflammatory Bowel Disease (IBD): Cause or Consequence? IBD Treatment Targeting the Gut Microbiome. Pathogens.

[4] Lloyd-Price J., Arze C., Ananthakrishnan A.N., Schirmer M., Avila-Pacheco J., Poon T.W., Andrews E., Ajami N.J., Bonham K.S., Brislawn C.J. (2019). Multi-omics of the gut microbial ecosystem in inflammatory bowel diseases. Nature.

[5] Gyriki D., Nikolaidis C., Stavropoulou E., Bezirtzoglou I., Tsigalou C., Vradelis S., Bezirtzoglou E. (2024). Exploring the Gut Microbiome’s Role in Inflammatory Bowel Disease: Insights and Interventions. J. Pers. Med..

[6] Kasahara K., Kerby R.L., Zhang Q., Pradhan M., Mehrabian M., Lusis A.J., Bergstrom G., Backhed F., Rey F.E. (2023). Gut bacterial metabolism contributes to host global purine homeostasis. Cell Host Microbe.

[7] Choden T., Cohen N.A. (2022). The gut microbiome and the immune system. Explor. Med..

[8] Wiertsema S.P., van Bergenhenegouwen J., Garssen J., Knippels L.M.J. (2021). The Interplay between the Gut Microbiome and the Immune System in the Context of Infectious Diseases throughout Life and the Role of Nutrition in Optimizing Treatment Strategies. Nutrients.

[9] Wu H.J., Wu E. (2012). The role of gut microbiota in immune homeostasis and autoimmunity. Gut Microbes.

[10] Blaak E.E., Canfora E.E., Theis S., Frost G., Groen A.K., Mithieux G., Nauta A., Scott K., Stahl B., van Harsselaar J. (2020). Short chain fatty acids in human gut and metabolic health. Benef. Microbes.

[11] Yang Q., Liang Q., Balakrishnan B., Belobrajdic D.P., Feng Q.J., Zhang W. (2020). Role of Dietary Nutrients in the Modulation of Gut Microbiota: A Narrative Review. Nutrients.

[12] Igai K., Itakura M., Nishijima S., Tsurumaru H., Suda W., Tsutaya T., Tomitsuka E., Tadokoro K., Baba J., Odani S. (2016). Nitrogen fixation and nifH diversity in human gut microbiota. Sci. Rep..

[13] Gonzalez-Soltero R., Bailen M., de Lucas B., Ramirez-Goercke M.I., Pareja-Galeano H., Larrosa M. (2020). Role of Oral and Gut Microbiota in Dietary Nitrate Metabolism and Its Impact on Sports Performance. Nutrients.

[14] Schimmel P., Stahl B., Knol J., Belzer C. (2023). The infant gut microbiota: In pursuit of non-protein nitrogen. Gut Microbes.

[15] Jian X., Zhu Y., Ouyang J., Wang Y., Lei Q., Xia J., Guan Y., Zhang J., Guo J., He Y. (2020). Alterations of gut microbiome accelerate multiple myeloma progression by increasing the relative abundances of nitrogen-recycling bacteria. Microbiome.

[16] Abdallah A., Elemba E., Zhong Q., Sun Z. (2020). Gastrointestinal Interaction between Dietary Amino Acids and Gut Microbiota: With Special Emphasis on Host Nutrition. Curr. Protein Pept. Sci..

[17] Diether N.E., Willing B.P. (2019). Microbial Fermentation of Dietary Protein: An Important Factor in Diet(-)Microbe(-)Host Interaction. Microorganisms.

[18] Ni J., Shen T.-C.D., Chen E.Z., Bittinger K., Bailey A., Roggiani M., Sirota-Madi A., Friedman E.S., Chau L., Lin A. (2017). A role for bacterial urease in gut dysbiosis and Crohn’s disease. Sci. Transl. Med..

[19] Andriamihaja M., Lan A., Beaumont M., Audebert M., Wong X., Yamada K., Yin Y., Tome D., Carrasco-Pozo C., Gotteland M. (2015). The deleterious metabolic and genotoxic effects of the bacterial metabolite p-cresol on colonic epithelial cells. Free Radic. Biol. Med..

[20] Calvez J., Azzout-Marniche D., Tome D. (2024). Protein quality, nutrition and health. Front. Nutr..

[21] Silk D.B., Fairclough P.D., Clark M.L., Hegarty J.E., Marrs T.C., Addison J.M., Burston D., Clegg K.M., Matthews D.M. (1980). Use of a peptide rather than free amino acid nitrogen source in chemically defined “elemental” diets. JPEN J. Parenter. Enter. Nutr..

[22] Bartlett A., Kleiner M. (2022). Dietary protein and the intestinal microbiota: An understudied relationship. iScience.

[23] Carlstrom M., Moretti C.H., Weitzberg E., Lundberg J.O. (2020). Microbiota, diet and the generation of reactive nitrogen compounds. Free Radic. Biol. Med..

[24] Wu S., Bhat Z.F., Gounder R.S., Mohamed Ahmed I.A., Al-Juhaimi F.Y., Ding Y., Bekhit A.E.A. (2022). Effect of Dietary Protein and Processing on Gut Microbiota-A Systematic Review. Nutrients.

[25] Duncan S.H., Iyer A., Russell W.R. (2021). Impact of protein on the composition and metabolism of the human gut microbiota and health. Proc. Nutr. Soc..

[26] Zhao J., Zhang X., Liu H., Brown M.A., Qiao S. (2019). Dietary Protein and Gut Microbiota Composition and Function. Curr. Protein Pept. Sci..

[27] Leeming E.R., Johnson A.J., Spector T.D., Le Roy C.I. (2019). Effect of Diet on the Gut Microbiota: Rethinking Intervention Duration. Nutrients.

[28] Chalvon-Demersay T., Luise D., Le Floc’h N., Tesseraud S., Lambert W., Bosi P., Trevisi P., Beaumont M., Corrent E. (2021). Functional Amino Acids in Pigs and Chickens: Implication for Gut Health. Front. Vet. Sci..

[29] Broer S. (2023). Intestinal Amino Acid Transport and Metabolic Health. Annu. Rev. Nutr..

[30] Kumar M., Tomar M., Punia S., Dhakane-Lad J., Dhumal S., Changan S., Senapathy M., Berwal M.K., Sampathrajan V., Sayed A.A.S. (2022). Plant-based proteins and their multifaceted industrial applications. LWT.

[31] Hertzler S.R., Lieblein-Boff J.C., Weiler M., Allgeier C. (2020). Plant Proteins: Assessing Their Nutritional Quality and Effects on Health and Physical Function. Nutrients.

[32] Nimni M.E., Han B., Cordoba F. (2007). Are we getting enough sulfur in our diet?. Nutr. Metab..

[33] US Department of Health and Human Services What are U.S. Standards and Regulations for Nitrates and Nitrites Exposure? Registry, Agency for Toxic Substances and Disease Registry. Centers for Disease Control and Prevention: 2013. https://archive.cdc.gov/www_atsdr_cdc_gov/csem/nitrate-nitrite/standards.html.

[34] Karwowska M., Kononiuk A. (2020). Nitrates/Nitrites in Food-Risk for Nitrosative Stress and Benefits. Antioxidants.

[35] Keller R.M., Beaver L., Prater M.C., Hord N.G. (2020). Dietary Nitrate and Nitrite Concentrations in Food Patterns and Dietary Supplements. Nutr. Today.

[36] Schlagenhauf U. (2022). On the Role of Dietary Nitrate in the Maintenance of Systemic and Oral Health. Dent. J..

[37] Kamalian A., Sohrabi Asl M., Dolatshahi M., Afshari K., Shamshiri S., Momeni Roudsari N., Momtaz S., Rahimi R., Abdollahi M., Abdolghaffari A.H. (2020). Interventions of natural and synthetic agents in inflammatory bowel disease, modulation of nitric oxide pathways. World J. Gastroenterol..

[38] Jakhar D., Sarin S.K., Kaur S. (2024). Gut microbiota and dynamics of ammonia metabolism in liver disease. npj Gut Liver.

[39] Barmore W., Azad F., Stone W.L. Physiology, Urea Cycle. https://www.ncbi.nlm.nih.gov/books/NBK513323/.

[40] Lapierre H., Lobley G.E. (2001). Nitrogen Recycling in the Ruminant: A Review. J. Dairy Sci..

[41] Wu G., Wu Z., Dai Z., Yang Y., Wang W., Liu C., Wang B., Wang J., Yin Y. (2013). Dietary requirements of “nutritionally non-essential amino acids” by animals and humans. Amino Acids.

[42] Ren W., Wang K., Yin J., Chen S., Liu G., Tan B., Wu G., Bazer F.W., Peng Y., Yin Y. (2016). Glutamine-Induced Secretion of Intestinal Secretory Immunoglobulin A: A Mechanistic Perspective. Front. Immunol..

[43] Perna S., Alalwan T.A., Alaali Z., Alnashaba T., Gasparri C., Infantino V., Hammad L., Riva A., Petrangolini G., Allegrini P. (2019). The Role of Glutamine in the Complex Interaction between Gut Microbiota and Health: A Narrative Review. Int. J. Mol. Sci..

[44] Wang W.W., Qiao S.Y., Li D.F. (2009). Amino acids and gut function. Amino Acids.

[45] Fotiadis C., Adamis S., Misiakos E.P., Genetzakis M., Antonakis P.T., Tsekouras D.K., Gorgoulis V.G., Zografos G.C., Papalois A., Fotinou M. (2007). The prophylactic effect of L-arginine in acute ischaemic colitis in a rat model of ischaemia/reperfusion injury. Acta Chir. Belg..

[46] Coburn L.A., Gong X., Singh K., Asim M., Scull B.P., Allaman M.M., Williams C.S., Rosen M.J., Washington M.K., Barry D.P. (2012). L-arginine supplementation improves responses to injury and inflammation in dextran sulfate sodium colitis. PLoS ONE.

[47] Beaumont M., Blachier F. (2020). Amino Acids in Intestinal Physiology and Health. Adv. Exp. Med. Biol..

[48] Kucharzik T., Stoll R., Lugering N., Domschke W. (1995). Circulating antiinflammatory cytokine IL-10 in patients with inflammatory bowel disease (IBD). Clin. Exp. Immunol..

[49] Ikeda Y., Matsuda S. (2023). Gut Protective Effect from D-Methionine or Butyric Acid against DSS and Carrageenan-Induced Ulcerative Colitis. Molecules.

[50] Hara T., Meng S., Motooka D., Sato H., Arao Y., Tsuji Y., Yabumoto T., Doki Y., Eguchi H., Uchida S. (2024). Fat and proteolysis due to methionine, tryptophan, and niacin deficiency leads to alterations in gut microbiota and immune modulation in inflammatory bowel disease. Cancer Sci..

[51] Liu G., Yu L., Fang J., Andy Hu C.-A., Yin J., Ni H., Ren W., Duraipandiyan V., Chen S., Abdullah Al-Dhabi N. (2017). Methionine restriction on oxidative stress and immune response in dss-induced colitis mice. Oncotarget.

[52] Liu Y., Wang X., Hu C.A. (2017). Therapeutic Potential of Amino Acids in Inflammatory Bowel Disease. Nutrients.

[53] Hord N.G., Tang Y., Bryan N.S. (2009). Food sources of nitrates and nitrites: The physiologic context for potential health benefits. Am. J. Clin. Nutr..

[54] Muro P., Zhang L., Li S., Zhao Z., Jin T., Mao F., Mao Z. (2024). The emerging role of oxidative stress in inflammatory bowel disease. Front. Endocrinol..

[55] Levine J.J., Pettei M.J., Valderrama E., Gold D.M., Kessler B.H., Trachtman H. (1998). Nitric oxide and inflammatory bowel disease: Evidence for local intestinal production in children with active colonic disease. J. Pediatr. Gastroenterol. Nutr..

[56] Cross R.K., Wilson K.T. (2003). Nitric oxide in inflammatory bowel disease. Inflamm. Bowel Dis..

[57] Baldelli V., Scaldaferri F., Putignani L., Del Chierico F. (2021). The Role of Enterobacteriaceae in Gut Microbiota Dysbiosis in Inflammatory Bowel Diseases. Microorganisms.

[58] Kostic A.D., Xavier R.J., Gevers D. (2014). The microbiome in inflammatory bowel disease: Current status and the future ahead. Gastroenterology.

[59] Beaumont M., Portune K.J., Steuer N., Lan A., Cerrudo V., Audebert M., Dumont F., Mancano G., Khodorova N., Andriamihaja M. (2017). Quantity and source of dietary protein influence metabolite production by gut microbiota and rectal mucosa gene expression: A randomized, parallel, double-blind trial in overweight humans. Am. J. Clin. Nutr..

[60] Konozy E.H.E., Osman M.E.M. (2024). From inflammation to immune regulation: The dual nature of dietary lectins in health and disease. Heliyon.

[61] Banwell J.G., Howard R., Kabir I., Costerton J.W. (1988). Bacterial overgrowth by indigenous microflora in the phytohemagglutinin-fed rat. Can. J. Microbiol..

[62] Greer F., Pusztai A. (1985). Toxicity of kidney bean (*Phaseolus vulgaris*) in rats: Changes in intestinal permeability. Digestion.

[63] Cohen L.J., Han S.M., Lau P., Guisado D., Liang Y., Nakashige T.G., Ali T., Chiang D., Rahman A., Brady S.F. (2022). Unraveling function and diversity of bacterial lectins in the human microbiome. Nat. Commun..

[64] Lee J.S., Wang R.X., Goldberg M.S., Clifford G.P., Kao D.J., Colgan S.P. (2020). Microbiota-Sourced Purines Support Wound Healing and Mucous Barrier Function. iScience.

[65] Chen T., Tao Y., Wang Q., Pei Y., Zhao Z., Yang W., Lu Y. (2024). Utilizing an integrated bioinformatics and machine learning approach to uncover biomarkers linking ulcerative colitis to purine metabolism-related genes. Heliyon.

[66] Worledge C.S., Kostelecky R.E., Zhou L., Bhagavatula G., Colgan S.P., Lee J.S. (2024). Allopurinol Disrupts Purine Metabolism to Increase Damage in Experimental Colitis. Cells.

[67] Swidsinski A., Loening-Baucke V., Theissig F., Engelhardt H., Bengmark S., Koch S., Lochs H., Dorffel Y. (2007). Comparative study of the intestinal mucus barrier in normal and inflamed colon. Gut.

[68] Wang Z., Li Y., Liao W., Huang J., Liu Y., Li Z., Tang J. (2022). Gut microbiota remodeling: A promising therapeutic strategy to confront hyperuricemia and gout. Front. Cell. Infect. Microbiol..

[69] Hamid O., Alsabbagh Alchirazi K., Eltelbany A., Nanah R., Regueiro M. (2023). Increased prevalence of gout in patients with inflammatory bowel disease: A population-based study. JGH Open.

[70] Li N., Lewis P., Samuelson D., Liboni K., Neu J. (2004). Glutamine regulates Caco-2 cell tight junction proteins. Am. J. Physiol. Gastrointest. Liver Physiol..

[71] Li N., Neu J. (2009). Glutamine deprivation alters intestinal tight junctions via a PI3-K/Akt mediated pathway in Caco-2 cells. J. Nutr..

[72] De-Souza D.A., Greene L.J. (2005). Intestinal permeability and systemic infections in critically ill patients: Effect of glutamine. Crit. Care Med..

[73] Li J.Y., Guo Y.C., Zhou H.F., Yue T.T., Wang F.X., Sun F., Wang W.Z. (2023). Arginine metabolism regulates the pathogenesis of inflammatory bowel disease. Nutr. Rev..

[74] Singh K., Coburn L.A., Barry D.P., Asim M., Scull B.P., Allaman M.M., Lewis N.D., Washington M.K., Rosen M.J., Williams C.S. (2013). Deletion of cationic amino acid transporter 2 exacerbates dextran sulfate sodium colitis and leads to an IL-17-predominant T cell response. Am. J. Physiol. Gastrointest. Liver Physiol..

[75] Hong S.K., Maltz B.E., Coburn L.A., Slaughter J.C., Chaturvedi R., Schwartz D.A., Wilson K.T. (2010). Increased serum levels of L-arginine in ulcerative colitis and correlation with disease severity. Inflamm. Bowel Dis..

[76] Coburn L.A., Horst S.N., Allaman M.M., Brown C.T., Williams C.S., Hodges M.E., Druce J.P., Beaulieu D.B., Schwartz D.A., Wilson K.T. (2016). L-Arginine Availability and Metabolism Is Altered in Ulcerative Colitis. Inflamm. Bowel Dis..

[77] Andrade M.E.R., Barros P.A.V.d., Menta P.L.d.R., Costa G.M.F., Miranda S.E.M., Leocádio P.C.L., Almeida-Leite C.M.d., Generoso S.d.V., Leite J.I.A., Cardoso V.N. (2019). Arginine supplementation reduces colonic injury, inflammation and oxidative stress of DSS-induced colitis in mice. J. Funct. Foods.

[78] Kruidenier L., Kuiper I., Lamers C.B., Verspaget H.W. (2003). Intestinal oxidative damage in inflammatory bowel disease: Semi-quantification, localization, and association with mucosal antioxidants. J. Pathol..

[79] Dudzinska E., Gryzinska M., Ognik K., Gil-Kulik P., Kocki J. (2018). Oxidative Stress and Effect of Treatment on the Oxidation Product Decomposition Processes in IBD. Oxid. Med. Cell Longev..

[80] Jahanian R. (2009). Immunological responses as affected by dietary protein and arginine concentrations in starting broiler chicks. Poult. Sci..

[81] Fernandez-Tome S., Ortega Moreno L., Chaparro M., Gisbert J.P. (2021). Gut Microbiota and Dietary Factors as Modulators of the Mucus Layer in Inflammatory Bowel Disease. Int. J. Mol. Sci..

[82] Blander J.M., Longman R.S., Iliev I.D., Sonnenberg G.F., Artis D. (2017). Regulation of inflammation by microbiota interactions with the host. Nat. Immunol..

[83] Fernandez-Tome S., Marin A.C., Ortega Moreno L., Baldan-Martin M., Mora-Gutierrez I., Lanas-Gimeno A., Moreno-Monteagudo J.A., Santander C., Sanchez B., Chaparro M. (2019). Immunomodulatory Effect of Gut Microbiota-Derived Bioactive Peptides on Human Immune System from Healthy Controls and Patients with Inflammatory Bowel Disease. Nutrients.

[84] Fernández-Tomé S., Hernández-Ledesma B., Chaparro M., Indiano-Romacho P., Bernardo D., Gisbert J.P. (2019). Role of food proteins and bioactive peptides in inflammatory bowel disease. Trends Food Sci. Technol..

[85] Colgan S.P., Curtis V.F., Campbell E.L. (2013). The inflammatory tissue microenvironment in IBD. Inflamm. Bowel Dis..

[86] Zhang C., Li Q., Xing J., Yang Y., Zhu M., Lin L., Yu Y., Cai X., Wang X. (2024). Tannic acid and zinc ion coordination of nanase for the treatment of inflammatory bowel disease by promoting mucosal repair and removing reactive oxygen and nitrogen species. Acta Biomater..

[87] Boughton-Smith N.K., Evans S.M., Hawkey C.J., Cole A.T., Balsitis M., Whittle B.J., Moncada S. (1993). Nitric oxide synthase activity in ulcerative colitis and Crohn’s disease. Lancet.

[88] Seril D.N., Liao J., Yang G.Y., Yang C.S. (2003). Oxidative stress and ulcerative colitis-associated carcinogenesis: Studies in humans and animal models. Carcinogenesis.

[89] Stettner N., Rosen C., Bernshtein B., Gur-Cohen S., Frug J., Silberman A., Sarver A., Carmel-Neiderman N.N., Eilam R., Biton I. (2018). Induction of Nitric-Oxide Metabolism in Enterocytes Alleviates Colitis and Inflammation-Associated Colon Cancer. Cell Rep..

[90] Abot A., Fried S., Cani P.D., Knauf C. (2022). Reactive Oxygen Species/Reactive Nitrogen Species as Messengers in the Gut: Impact on Physiology and Metabolic Disorders. Antioxid. Redox Signal..

[91] Linares R., Frances R., Gutierrez A., Juanola O. (2021). Bacterial Translocation as Inflammatory Driver in Crohn’s Disease. Front. Cell Dev. Biol..

[92] Stummer N., Feichtinger R.G., Weghuber D., Kofler B., Schneider A.M. (2023). Role of Hydrogen Sulfide in Inflammatory Bowel Disease. Antioxidants.

[93] Moon J.Y., Kye B.H., Ko S.H., Yoo R.N. (2023). Sulfur Metabolism of the Gut Microbiome and Colorectal Cancer: The Threat to the Younger Generation. Nutrients.

[94] Beaumont M., Andriamihaja M., Lan A., Khodorova N., Audebert M., Blouin J.M., Grauso M., Lancha L., Benetti P.H., Benamouzig R. (2016). Detrimental effects for colonocytes of an increased exposure to luminal hydrogen sulfide: The adaptive response. Free Radic. Biol. Med..

[95] Blachier F., Andriamihaja M., Larraufie P., Ahn E., Lan A., Kim E. (2021). Production of hydrogen sulfide by the intestinal microbiota and epithelial cells and consequences for the colonic and rectal mucosa. Am. J. Physiol. Gastrointest. Liver Physiol..

[96] Braccia D.J., Jiang X., Pop M., Hall A.B. (2021). The Capacity to Produce Hydrogen Sulfide (H(2)S) via Cysteine Degradation Is Ubiquitous in the Human Gut Microbiome. Front. Microbiol..

[97] Cremin J.D., Fitch M.D., Fleming S.E. (2003). Glucose alleviates ammonia-induced inhibition of short-chain fatty acid metabolism in rat colonic epithelial cells. Am. J. Physiol. Gastrointest. Liver Physiol..

[98] Andriamihaja M., Davila A.M., Eklou-Lawson M., Petit N., Delpal S., Allek F., Blais A., Delteil C., Tome D., Blachier F. (2010). Colon luminal content and epithelial cell morphology are markedly modified in rats fed with a high-protein diet. Am. J. Physiol. Gastrointest. Liver Physiol..

[99] Blachier F., Andriamihaja M. (2022). Effects of the L-tyrosine-derived bacterial metabolite p-cresol on colonic and peripheral cells. Amino Acids.

[100] Toft P.B., Vanslette A.M., Trost K., Moritz T., Gillum M.P., Backhed F., Arora T. (2023). Microbial metabolite p-cresol inhibits gut hormone expression and regulates small intestinal transit in mice. Front. Endocrinol..

[101] Al Hinai E.A., Kullamethee P., Rowland I.R., Swann J., Walton G.E., Commane D.M. (2019). Modelling the role of microbial p-cresol in colorectal genotoxicity. Gut Microbes.

[102] Mimoun S., Andriamihaja M., Chaumontet C., Atanasiu C., Benamouzig R., Blouin J.M., Tome D., Bouillaud F., Blachier F. (2012). Detoxification of H_2_S by differentiated colonic epithelial cells: Implication of the sulfide oxidizing unit and of the cell respiratory capacity. Antioxid. Redox Signal..

[103] Blachier F., Beaumont M., Andriamihaja M., Davila A.M., Lan A., Grauso M., Armand L., Benamouzig R., Tome D. (2017). Changes in the Luminal Environment of the Colonic Epithelial Cells and Physiopathological Consequences. Am. J. Pathol..

[104] De Preter V., Arijs I., Windey K., Vanhove W., Vermeire S., Schuit F., Rutgeerts P., Verbeke K. (2012). Decreased mucosal sulfide detoxification is related to an impaired butyrate oxidation in ulcerative colitis. Inflamm. Bowel Dis..

[105] Kou Y., Zhang S., Chen J., Shen Y., Zhang Z., Huang H., Ma Y., Xiang Y., Liao L., Zhou J. (2024). A mouse protozoan boosts antigen-specific mucosal IgA responses in a specific lipid metabolism- and signaling-dependent manner. Nat. Commun..

[106] Liu Y., Rhoads J.M. (2013). Communication between B-Cells and Microbiota for the Maintenance of Intestinal Homeostasis. Antibodies.

[107] Takeuchi T., Ohno H. (2022). IgA in human health and diseases: Potential regulator of commensal microbiota. Front. Immunol..

[108] Cruzat V., Macedo Rogero M., Noel Keane K., Curi R., Newsholme P. (2018). Glutamine: Metabolism and Immune Function, Supplementation and Clinical Translation. Nutrients.

[109] Brown K.A., Back S.J., Ruchelli E.D., Markowitz J., Mascarenhas M., Verma R., Piccoli D.A., Baldassano R.N. (2002). Lamina propria and circulating interleukin-6 in newly diagnosed pediatric inflammatory bowel disease patients. Am. J. Gastroenterol..

[110] Xue H., Sufit A.J., Wischmeyer P.E. (2011). Glutamine therapy improves outcome of in vitro and in vivo experimental colitis models. JPEN J. Parenter. Enter. Nutr..

[111] Katinios G., Casado-Bedmar M., Walter S.A., Vicario M., Gonzalez-Castro A.M., Bednarska O., Soderholm J.D., Hjortswang H., Keita A.V. (2020). Increased Colonic Epithelial Permeability and Mucosal Eosinophilia in Ulcerative Colitis in Remission Compared With Irritable Bowel Syndrome and Health. Inflamm. Bowel Dis..

[112] Sultan S., El-Mowafy M., Elgaml A., Ahmed T.A.E., Hassan H., Mottawea W. (2021). Metabolic Influences of Gut Microbiota Dysbiosis on Inflammatory Bowel Disease. Front. Physiol..

[113] Michielan A., D’Inca R. (2015). Intestinal Permeability in Inflammatory Bowel Disease: Pathogenesis, Clinical Evaluation, and Therapy of Leaky Gut. Mediat. Inflamm..

[114] Reese A.T., Pereira F.C., Schintlmeister A., Berry D., Wagner M., Hale L.P., Wu A., Jiang S., Durand H.K., Zhou X. (2018). Microbial nitrogen limitation in the mammalian large intestine. Nat. Microbiol..

[115] Holmes A.J., Chew Y.V., Colakoglu F., Cliff J.B., Klaassens E., Read M.N., Solon-Biet S.M., McMahon A.C., Cogger V.C., Ruohonen K. (2017). Diet-Microbiome Interactions in Health Are Controlled by Intestinal Nitrogen Source Constraints. Cell Metab..

[116] De Angelis M., Ferrocino I., Calabrese F.M., De Filippis F., Cavallo N., Siragusa S., Rampelli S., Di Cagno R., Rantsiou K., Vannini L. (2020). Diet influences the functions of the human intestinal microbiome. Sci. Rep..

[117] Seidelmann S.B., Claggett B., Cheng S., Henglin M., Shah A., Steffen L.M., Folsom A.R., Rimm E.B., Willett W.C., Solomon S.D. (2018). Dietary carbohydrate intake and mortality: A prospective cohort study and meta-analysis. Lancet Public Health.

[118] Leclerc M., Bedu-Ferrari C., Etienne-Mesmin L., Mariadassou M., Lebreuilly L., Tran S.L., Brazeau L., Mayeur C., Delmas J., Rue O. (2021). Nitric Oxide Impacts Human Gut Microbiota Diversity and Functionalities. mSystems.

[119] Davila A.M., Blachier F., Gotteland M., Andriamihaja M., Benetti P.H., Sanz Y., Tome D. (2013). Intestinal luminal nitrogen metabolism: Role of the gut microbiota and consequences for the host. Pharmacol. Res..

[120] Prindiville T.P., Sheikh R.A., Cohen S.H., Tang Y.J., Cantrell M.C., Silva J. (2000). Bacteroides fragilis enterotoxin gene sequences in patients with inflammatory bowel disease. Emerg. Infect. Dis..

[121] Steck N., Hoffmann M., Sava I.G., Kim S.C., Hahne H., Tonkonogy S.L., Mair K., Krueger D., Pruteanu M., Shanahan F. (2011). Enterococcus faecalis Metalloprotease Compromises Epithelial Barrier and Contributes to Intestinal Inflammation. Gastroenterology.

[122] Cai J., Chen Z., Wu W., Lin Q., Liang Y. (2022). High animal protein diet and gut microbiota in human health. Crit. Rev. Food Sci. Nutr..

[123] Tiso M., Schechter A.N. (2015). Nitrate reduction to nitrite, nitric oxide and ammonia by gut bacteria under physiological conditions. PLoS ONE.

[124] Wu L., Tang Z., Chen H., Ren Z., Ding Q., Liang K., Sun Z. (2021). Mutual interaction between gut microbiota and protein/amino acid metabolism for host mucosal immunity and health. Anim. Nutr..

[125] Schanuel C., Dias E., Ferreira A., Bertges K., Bertges L. (2019). Glutamine as A Therapeutic Strategy in Inflammatory Bowel Diseases: A Systematic Review. Gastroenterol. Hepatol. Dig. Disord..

[126] Singh K., Gobert A.P., Coburn L.A., Barry D.P., Allaman M., Asim M., Luis P.B., Schneider C., Milne G.L., Boone H.H. (2019). Dietary Arginine Regulates Severity of Experimental Colitis and Affects the Colonic Microbiome. Front. Cell. Infect. Microbiol..

[127] Nciri N., Cho N., Bergaoui N., Mhamdi F.E., Ammar A.B., Trabelsi N., Zekri S., Guémira F., Mansour A.B., Sassi F.H. (2015). Effect of White Kidney Beans (*Phaseolus vulgaris* L. var. Beldia) on Small Intestine Morphology and Function in Wistar Rats. J. Med. Food.

[128] Kordás K., Szalmay G., Bardocz S., Pusztai Á., Varga G. (2001). Phytohaemagglutinin inhibits gastric acid but not pepsin secretion in conscious rats. J. Physiol.-Paris.

[129] Lucius K. (2020). Dietary Lectins: Gastrointestinal and Immune Effects. Altern. Complement. Ther..

[130] Ungaro R., Mehandru S., Allen P.B., Peyrin-Biroulet L., Colombel J.F. (2017). Ulcerative colitis. Lancet.

[131] Lavelle A., Sokol H. (2020). Gut microbiota-derived metabolites as key actors in inflammatory bowel disease. Nat. Rev. Gastroenterol. Hepatol..

[132] Wellens J., Vissers E., Matthys C., Vermeire S., Sabino J. (2023). Personalized Dietary Regimens for Inflammatory Bowel Disease: Current Knowledge and Future Perspectives. Pharmgenom. Pers. Med..

[133] Biesiekierski J.R., Jalanka J., Staudacher H.M. (2019). Can Gut Microbiota Composition Predict Response to Dietary Treatments?. Nutrients.

[134] Olendzki B., Bucci V., Cawley C., Maserati R., McManus M., Olednzki E., Madziar C., Chiang D., Ward D.V., Pellish R. (2022). Dietary manipulation of the gut microbiome in inflammatory bowel disease patients: Pilot study. Gut Microbes.

[135] Aleksandrova K., Romero-Mosquera B., Hernandez V. (2017). Diet, Gut Microbiome and Epigenetics: Emerging Links with Inflammatory Bowel Diseases and Prospects for Management and Prevention. Nutrients.

[136] Tosti V., Bertozzi B., Fontana L. (2018). Health Benefits of the Mediterranean Diet: Metabolic and Molecular Mechanisms. J. Gerontol. A Biol. Sci. Med. Sci..

[137] De Filippis F., Pellegrini N., Vannini L., Jeffery I.B., La Storia A., Laghi L., Serrazanetti D.I., Di Cagno R., Ferrocino I., Lazzi C. (2016). High-level adherence to a Mediterranean diet beneficially impacts the gut microbiota and associated metabolome. Gut.

[138] Haskey N., Estaki M., Ye J., Shim R.K., Singh S., Dieleman L.A., Jacobson K., Gibson D.L. (2023). A Mediterranean Diet Pattern Improves Intestinal Inflammation Concomitant with Reshaping of the Bacteriome in Ulcerative Colitis: A Randomised Controlled Trial. J. Crohns Colitis.

[139] Eom T., Kim Y.S., Choi C.H., Sadowsky M.J., Unno T. (2018). Current understanding of microbiota- and dietary-therapies for treating inflammatory bowel disease. J. Microbiol..

